# Hemodynamic Differences between Patients Hospitalized with Acutely Decompensated Chronic Heart Failure and De Novo Heart Failure

**DOI:** 10.3390/jcm12216768

**Published:** 2023-10-26

**Authors:** Agata Galas, Paweł Krzesiński, Małgorzata Banak, Grzegorz Gielerak

**Affiliations:** Department of Cardiology and Internal Diseases, Military Institute of Medicine—National Research Institute, 04-141 Warsaw, Poland; pkrzesinski@wim.mil.pl (P.K.); mbanak@wim.mil.pl (M.B.); ggielerak@wim.mil.pl (G.G.)

**Keywords:** acute heart failure, de novo heart failure, impedance cardiography, congestion, thoracic fluid content, hypervolemia, natriuretic peptide

## Abstract

Background: Heart failure (HF) is associated with high mortality, morbidity, and frequent hospitalizations due to acute HF (AHF) and requires immediate diagnosis and individualized therapy. Some differences between acutely decompensated chronic heart failure (ADCHF) and de novo HF (dnHF) patients in terms of clinical profile, comorbidities, and outcomes have been previously identified, but the hemodynamics related to both of these clinical states are still not well recognized. Purpose: To compare patients hospitalized with ADCHF to those with dnHF, with a special emphasis on hemodynamic profiles at admission and changes due to hospital treatment. Methods: This study enrolled patients who were at least 18 years old, hospitalized due to AHF (both ADCHF and dnHF), and who underwent detailed assessments at admission and at discharge. The patients’ hemodynamic profiles were assessed by impedance cardiography (ICG) and characterized in terms of heart rate (HR), blood pressure (BP), systemic vascular resistance index (SVRI), cardiac index (CI), stroke index (SI), and thoracic fluid content (TFC). Results: The study population consisted of 102 patients, most of whom were men (76.5%), with a mean left ventricle ejection fraction (LVEF) of 37.3 ± 14.1%. The dnHF patients were younger than the ADCHF group and more frequently presented with palpitations (*p* = 0.041) and peripheral hypoperfusion (*p* = 0.011). In terms of hemodynamics, dnHF was distinguished by higher HR (*p* = 0.029), diastolic BP (*p* = 0.029), SVRI (*p* = 0.013), and TFC (only numeric, *p* = 0.194) but lower SI (*p* = 0.043). The effect of hospital treatment on TFC was more pronounced in dnHF than in ADCHF, and this was also true of N-terminal pro-brain natriuretic peptide (NT-proBNP) and body mass. Some intergroup differences in the hemodynamic profile observed at admission persisted until discharge: higher HR (*p* = 0.002) and SVRI (trend, *p* = 0.087) but lower SI (*p* < 0.001) and CI (*p* = 0.023) in the dnHF group. Conclusions: In comparison to ADCHF, dnHF is associated with greater tachycardia, vasoconstriction, depressed cardiac performance, and congestion. Despite more effective diuretic therapy, other unfavorable hemodynamic features may still be present in dnHF patients at discharge.

## 1. Introduction

Heart failure (HF) remains a leading cause of morbidity, mortality, and hospitalization, mostly due to acute HF (AHF), which is a life-threatening condition [1,2]. Many classifications of AHF have been proposed based on hemodynamic profiles, etiology, or a history of prior HF diagnosis. Some experts postulate that AHF should include only patients with de novo HF (dnHF) and that other cases should be treated as a separate category (e.g., worsening of chronic HF) [3,4]. There are some arguments for this opinion. Patients with dnHF are supposed to be at the beginning of their HF “path” [5] and are mostly free from the implications of hemodynamic and metabolic disorders related to long-lasting inadequate cardiac output. Some differences between acute decompensated heart failure (ADCHF) and dnHF have been previously identified with regard to clinical profile [6], biomarkers [7], and outcomes [6,7]. According to the registries, dnHF patients are younger, present fewer comorbidities, and have a better one-year prognosis [7]. However, some contrary observations have only added to the controversies in this area [8].

The vast majority (90%) of AHF cases are caused by volume overload, and the main way to achieve decongestion is through diuretic therapy [1,2]. However, the most common factors precipitating dnHF are hypertensive heart disease and acute coronary syndrome (ACS), while in ADCHF, it is infection [6,9,10]. The differences in hemodynamic profiles may, therefore, be expected, and their identification seems to be crucial for optimizing therapeutic decisions [4,9]. Unfortunately, knowledge of the differences in the hemodynamic profiles and treatment effects based on previously diagnosed HF is still scarce. Some non-invasive tools, such as impedance cardiography (ICG), which has been revealed to have diagnostic value in different clinical settings in HF patients [11,12,13], could provide these relevant data.

Therefore, we aimed to compare patients hospitalized with ADCHF and dnHF, with a special emphasis on hemodynamic characteristics at admission and their changes due to hospital treatment.

## 2. Materials and Methods

This prospective observational study enrolled adult patients of both sexes hospitalized at the Department of Cardiology and Internal Diseases of the Military Institute of Medicine between November 2014 and March 2017 whose cause of hospitalization was AHF (defined according to the European Society of Cardiology guidelines [1,2]) and who required intravenous diuretic therapy. For the purpose of this analysis, patients were categorized into two groups: (1) dnHF, defined as those without a history of pre-existing HF, and (2) ADCHF, defined as an exacerbation of symptoms of previously diagnosed HF.

The exclusion criteria were described in our previous papers [11,13]. Briefly, patients with unstable angina, history of acute coronary syndrome and/or coronary artery bypass grafting surgery within the last 12 weeks, non-cardiogenic shock (i.e., sepsis or bleeding with hypotension requiring catecholamines), severe pulmonary hypertension or other severe lung conditions, pulmonary embolism, poorly controlled hypertension, acute and/or decompensated non-cardiovascular disease, valvular disease or other acquired heart defects requiring surgical intervention, anemia (hemoglobin < 10.0 g/dL), end-stage chronic kidney disease, and neoplastic disease were not eligible. The study protocol was approved by the local bioethics committee (Bioethics Committee of the Military Medical Institute in Warsaw, Warsaw, Poland, approval no. 14/WIM/2012; 16 May 2012), and all study participants provided their written informed consent. The study was registered at ClinicalTrials.gov (NCT 02355769). The patients were treated according to the current guidelines.

To assess the patients’ hemodynamic profiles, impedance cardiography (ICG, Niccomo™ device (Medis, Ilmenau, Germany)) was used. All ICG measurements were performed within 24 h of admission after 10 min of rest in a sitting position. ICG is a non-invasive technology measuring total electrical conductivity of the thorax and its changes in time. It detects the impedance changes caused by a high-frequency, low-magnitude current flowing through the thorax between 2 additional pairs of electrodes located outside of the measured segment. The sensing electrodes also detect the ECG signal. This examination allows the calculation of hemodynamic parameters, such as stroke volume (SV), stroke index (SI), cardiac index (CI), cardiac output (CO), ventricular ejection time (LVET), systemic vascular resistance index (SVRI), and thoracic fluid content (TFC). TFC is an especially important parameter as it indicates the extent of fluid accumulation in the chest, which could be useful in AHF as a good marker of congestion [11,14]. The advantage of this method is that it is inexpensive and can be performed bedside to assess parameters. ICG is useful in differentiating the causes of dyspnea in emergency settings [15] and in predicting HF decompensation [16]. The calculations of the parameters were presented in our previous paper [11,13]. Echocardiography was conducted using Vivid S6 (GE-Healthcare, Chicago, IL, USA) and Vivid 7 (GE-Healthcare, Chicago, IL, USA) ultrasound systems. The standard assessment included left ventricular ejection fraction (LVEF). There was no defined time limit for echocardiography, but the median time delay from admission to echocardiography was 3 days. Laboratory tests from peripheral venous blood samples were collected twice: within 2 h from admission and in the morning on the discharge day. The analysis included the levels of N-terminal pro-brain natriuretic peptide (NTproBNP), high-sensitivity troponin T (hsTnT), hemoglobin, white blood cell count (WBC), creatinine, and estimated glomerular filtration rate (eGFR), as calculated using the Modification of Diet in Renal Disease (MDRD) equation [17].

The statistical analysis was performed using Statistica 12.0 (StatSoft, Inc., Tulsa, OK, USA). The distribution and normality of the data were assessed via visual inspection and the Kolmogorov–Smirnov test. Continuous variables were presented as mean ± standard deviation (SD) for normal distribution and median (Me) and interquartile range (IQR) for non-normal distribution. Categorical variables were presented as absolute and relative frequencies (percentages). The change in selected variables was calculated as d_X (delta) = [absolute value at discharge] − [absolute value at admission]. For comparative analysis, the study group was stratified into dnHF and ADCHF groups. These subgroups were compared in terms of clinical, laboratory, and hemodynamic parameters using Student’s *t*-test or Mann–Whitney U-test for continuous variables and chi-squared or Fisher’s exact test for categorical variables. The values of selected continuous variables at admission and discharge were compared using Student’s *t*-test or Wilcoxon matched-pairs test. A *p*-value of <0.05 was considered statistically significant.

## 3. Results

### 3.1. Study Group Baseline Characteristics

The study population consisted of 102 patients, of whom 78 (76.5%) were male, with a mean left ventricle ejection fraction (LVEF) of 37.3 ± 14.1%. The majority of patients presented with symptoms of NYHA class III severity (n = 66, 64.7%), whereas the remaining 36 patients (35.3%) had symptoms of NYHA class IV severity. The most commonly reported symptoms were dyspnea on exertion, orthopnea, and edema (Table 1). Ischemic heart disease, hypertension, atrial fibrillation, and valvular heart disease were the most frequent concomitant diseases (Table 1). Most patients were treated according to the guidelines [1] with angiotensin-converting enzyme inhibitors (ACEIs) or angiotensin receptor blockers (ARBs) (70.6%), mineralocorticoid receptor antagonists (MRAs) (32.4%), beta-blockers (76.5%), and diuretics (72.5%). The demographic, baseline, and laboratory tests at admission are summarized in Table 1.

### 3.2. Comparison of Admission Characteristics of Patients Hospitalized with Acutely Decompensated CHF and De Novo HF

Among the study population, 27 patients (26.5%) presented with dnHF. These patients with dnHF were younger than the ADCHF patients (*p* = 0.012, Table 2). Thirty-nine (52%) ADCHF patients had been previously hospitalized due to HF decompensation. Before hospitalization, they less frequently received ACEIs (*p* = 0.0008), beta-blockers (*p* = 0.0001), MRAs (*p* = 0.007), and diuretics (*p* < 0.0001) (Table 2). Compared to ADCHF patients, dnHF patients more frequently presented with palpitations (*p* = 0.041) and peripheral hypoperfusion (*p* = 0.011). Conversely, ADCHF patients had a higher prevalence of atrial fibrillation in anamnesis (*p* = 0.017) and chronic kidney disease (CKD) (*p* = 0.014).

For the laboratory tests, patients with a first episode of AHF had higher WBC (*p* = 0.001), aspartate aminotransferase (AST; *p* = 0.037), hemoglobin (*p* = 0.004), and better kidney function, as evaluated by creatinine concentration (*p* = 0.03) and eGFR (*p* = 0.007). No significant differences in NT-proBNP and hsTnT were observed.

The most distinctive differences were identified in ICG hemodynamics. In comparison to the ADCHF group, dnHF patients were distinguished by a higher HR (*p* = 0.029), diastolic BP (DBP; *p* = 0.029), SVRI (*p* = 0.013), TFC (*p* = 0.19), and lower SI (*p* = 0.043) (Table 2). There was no intergroup difference in LVEF.

### 3.3. The Effect of Treatment in Patients Hospitalized with Acutely Decompensated CHF and De Novo HF

**Figure 1:**
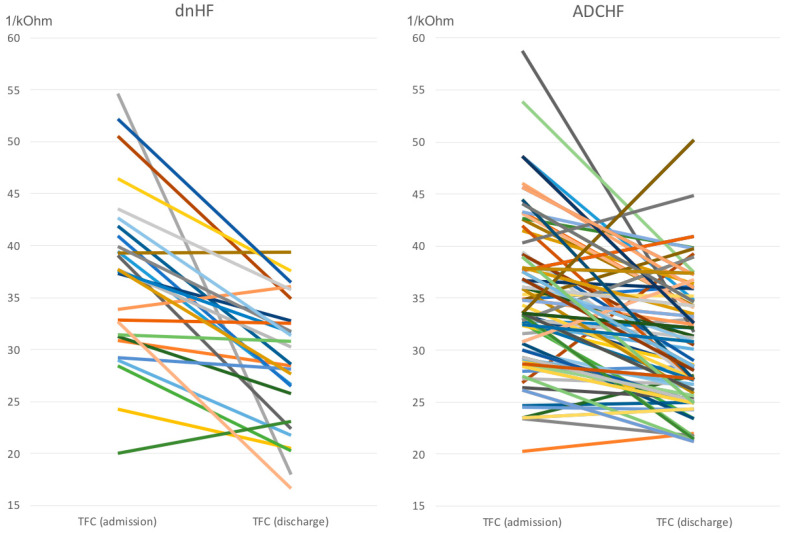
Individual changes of TFC in acutely decompensated CHF (ADCHF) and de novo HF (dnHF). Each color represents individual patient.

The effects of the applied treatment were expressed more in dnHF than in ADCHF (Table 3). The differences were consistently evident for TFC, which decreased from mean 37.3 to 28.8 1*kΩ^−1^ (*p* < 0.0001) in dnHF and from 35.0 to 30.6 1*kΩ^−1^ (*p* < 0.0001) in ADCHF (Table 3, Figure 1). They were also consistent in the NT-proBNP values, which decreased from mean 5648 to 1810 pg/mL (*p* < 0.000 1) in dnHF and from 5054 to 3095 pg/mL (*p* < 0.0001) in ADCHF, as well as in body mass, which decreased from 89.7 to 83.3 kg (*p* < 0.0001) in dnHF and from 87.2 to 82.9 kg (*p* < 0.0001) in ADCHF. Some intergroup differences in hemodynamic profiles observed at admission persisted until discharge; these included higher HR, higher SVRI, and lower SI in dnHF subjects (Table 3). The medicine regimen at discharge was similar for both groups. It should be mentioned that three patients, all from the ADCHF group, died while hospitalized.

## 4. Discussion

Our findings support the opinion that there are significant differences between dnHF and ADCHF patients. We identified the hemodynamic features that distinguish dnHF from ADCHF, which also correspond with discrepancies in symptoms. We also objectively depicted the different trajectories of hemodynamic changes in the course of hospitalization between the groups. In most studies of AHF, dnHF constitutes approximately 50% of the patient population [18], while in our study, it was 26%. However, in the EuroHeart Survey II (EHFS II), two-thirds of all patients with AHF requiring hospitalization already had a known history of HF [19]. This low prevalence of dnHF in our group may be explained by our exclusion of patients with ACS. Patients with dnHF were younger and less burdened with comorbidities. This could be explained by shorter exposure to long-lasting hemodynamic disorders related to chronic HF [3]. In terms of symptoms and signs, the dnHF group presented a greater intensity of dyspnea at rest, which is consistent with the study by Senni et al., which showed that patients with dnHF needed therapy more frequently in the intensive care unit than patients with worsening (but pre-existing) HF [20]. In our study, this phenomenon may be at least partly explained by the trend toward a higher admission TFC. In our previous paper, we proved that TFC is a useful diagnostic test both during hospital admission and while monitoring the effects of decongestion treatment [11]. Furthermore, it is worth mentioning that TFC is higher in patients with anemia [13]. Other clinical observations were also depicted in hemodynamic profiles. The higher incidence of peripheral hypoperfusion in dnHF was consistent with lower SI from ICG assessment. Additionally, the higher frequency of palpitations corresponded with higher HR. This tachycardia is probably a compensatory mechanism for maintaining cardiac output while maintaining low SI. In the dnHF group, we observed less frequent administration of guideline-recommended medicines (ACEIs, MRAs, ARBs, and diuretics), which is in accordance with the observation by Nawrocka-Millward et al. [7]. However, there are also reports of the opposite, such as that of Choi et al., which showed that ADCHF patients were less likely to be treated with ACEIs, ARBs, and beta-blockers [20]. The ADCHF group had a higher prevalence of atrial fibrillation, chronic kidney disease, and anemia. All these comorbidities might be related to long-lasting hemodynamic and inflammatory disorders related to chronic HF. Surprisingly, although it is believed that one of the most common reasons for worsening HF is infection [6,9,10,21], the WBC level was lower among ADCHF patients. In our study, no significant intergroup differences in NT-proBNP levels were observed. This is contrary to previous data showing higher levels of natriuretic peptides in ADCHF patients [6,7,10,20,22]. However, in the Korean Acute Heart Failure Registry [22], the dnHF group had significantly lower NT-proBNP levels despite having a similar severity of symptoms as the ADCHF group. It is also worth commenting that patients with dnHF presented with higher DBP and especially high SVRI. This finding supports previous reports of hypertension as a precipitating factor of AHF in the dnHF group. Similarly, in the FINN-AKVA study, it was reported that dnHF patients had higher SBP, DBP, and HR [23]. Our observations regarding hemodynamic changes as a result of treatment were also interesting. In the dnHF group, we observed more effective decongestion, depicted by a significant reduction in TFC, which was supported by a more distinctive decrease in NT-proBNP and body mass. This can be explained by the fact that most dnHF patients are diuretic naïve, with a lower probability of developing diuretic resistance [24]. The higher prevalence of chronic kidney disease in ADCHF patients may also have contributed to this effect. These findings are consistent with observations by Greene et al., who showed that dnHF patients experienced dyspnea relief more quickly [25]. A significantly higher reduction of NT-proBNP in dnHF patients has also been previously observed [7]. In addition, it is worth emphasizing that some hemodynamic parameters distinguishing dnHF from ADCHF patients at admission were still present at discharge. While the differences in HR, BP, and SVRI were slightly reduced, the disparities in SI and CI conversely increased. This effect draws attention to the potential adverse effects of the more pronounced decongestion noted in dnHF. The prognostic value of this phenomenon may be clinically relevant and underscores the need for careful post-discharge follow-up of dnHF patients. Importantly, the reported differences did not affect the length of hospitalization, which was similar in dnHF and ADCHF, as in the previous study [7]. Our work may be particularly important due to the global increase in HF incidence during the COVID-19 pandemic, including dnHF. In a study by Huang et al., 34.5% of patients with COVID-19 treated in an intensive care unit presented with left or right systolic dysfunction [26,27].

### 4.1. Clinical Implications

Our findings confirm that patients with dnHF and ADCHF are different not only in terms of clinical profile and outcomes but also, and maybe mainly, in hemodynamic profile. The strength of our study is the dynamic evaluation of hemodynamics from admission to discharge. Our results highlight the importance of individualized therapy based on hemodynamic or objective volume assessment. We also showed that patients with dnHF are more responsive to diuretic therapy. However, this positive feature, from the perspective of decongestion, can potentially negatively affect intravascular volume and cardiac output. All of these aspects should be considered during hospitalization and outpatient follow-up after discharge.

### 4.2. Study Limitations

The findings of our study should be interpreted in the context of a few limitations. The main limitation is the small size of the evaluated subgroups, which could have influenced the statistical power of some comparisons. The fact that the study was a single-center, observational one should also be considered a potential limitation.

Another important limitation is the exclusion of patients with ACS who present with dnHF. It is important to highlight that there was a 24 h window in which ICG assessments were performed, as the hemodynamic profile may change within less than an hour of initiating diuretic treatment. There was also no time limit to perform echocardiography (though the median was 3 days). We would also like to emphasize that it is only the fluid content within the thorax that can be measured by ICG, which significantly restricts any possible extrapolations regarding overall body fluid content. Finally, the lack of follow-up after discharge makes it impossible to draw connections between the differences in hemodynamic parameters and long-term prognosis.

## 5. Conclusions

The diagnosis of acute HF covers a heterogeneous group of patients. In comparison to ADCHF, dnHF more frequently presents with tachycardia, vasoconstriction, depressed cardiac performance, and congestion. Despite more effective diuretic action, other unfavorable hemodynamic features may still be present at discharge in patients with dnHF.

## Figures and Tables

**Table 1:** Baseline characteristics of the study group (n = 102).

Parameter	Study Group n = 102
Age	71.4 ± 12.5
Males	78 (76.5%)
HR (bpm)	87 ± 24
Systolic BP (mmHg)	135 ± 27
Diastolic BP (mmHg)	82 ± 14
De novo HF	27 (26.5%)
**Symptoms and signs, n (%)**	
Dyspnea at rest	41 (40.2%)
Dyspnea on effort	100 (98.1%)
Orthopnea	78 (77.2%)
Paroxysmal nocturnal dyspnea	44 (43.1%)
Chest pain	25 (24.5%)
Palpitations	33 (32.4%)
Edema	77 (75.5%)
Tachypnea	21 (20.6%)
Ascites	16 (15.7%)
Peripheral hypoperfusion	10 (9.8%)
Hepatomegaly	18 (17.6%)
**Concomitant disease, n (%)**	
Prior myocardial infarction	42 (41.1%)
Hypertension	68 (66.6%)
Atrial fibrillation	54 (52.9%)
Previous stroke or transient ischemic attack	9 (8.8%)
Diabetes mellitus (type 2)	50 (49.0%)
Chronic obstructive pulmonary disease	15 (14.7%)
Chronic kidney disease (stage ≥ 3)	30 (29.4%)
**Laboratory data on admission, mean ± SD**	
NT-proBNP (pg/mL)	6197 ± 7057
Creatinine (mg/dL)	1.31 ± 0.51
eGFR (mL/min/1.73 m^2^)	62.2 ± 23.9
Hemoglobin (g/dL)	12.6 ± 2.6

eGFR—estimated glomerular filtration rate, HF—heart failure, HR—heart rate, MI—myocardial infarction, NTproBNP—N-terminal pro-brain natriuretic peptide. Data presented as “mean + SD” or “n (%)”.

**Table 2:** Comparison of admission characteristics between patients hospitalized with acutely decompensated CHF (ADCHF) and de novo HF (dnHF).

Parameter	ADCHF Mean ± SD or Median (IQR)/n (%)	dnHF Mean ± SD or Median (IQR)/n (%)	p
Age (years)	75 (65–83)	68 (57–75)	0.012
Female	19 (25.3%)	5 (18.5%)	0.474
BMI (kg/m^2^)	29.9 ± 5.8	30.6 ± 8.3	0.834
**Symptoms and signs**			
NYHA class			
Class III	52 (69.3%)	14 (51.9%)	0.103
Class IV	23 (30.7%)	13 (48.1%)	
Dyspnea at rest	26 (34.7%)	15 (55.6%)	0.058
Dyspnea on effort	75 (100%)	25 (92.6%)	0.017
Orthopnea	55 (74.3%)	23 (85.2%)	0.249
Paroxysmal nocturnal dyspnea	32 (42.7%)	12 (44.4%)	0.873
Chest pain	17 (21.3%)	9 (33.3%)	0.214
Palpitations	20 (26.7%)	13 (48.2%)	0.041
Edema	62 (82.7%)	18 (66.7%)	0.083
Tachypnea	16 (21.3%)	5 (18.5%)	0.756
Ascites	11 (14.7%)	5 (18.5%)	0.637
Peripheral hypoperfusion	4 (5.3%)	6 (22.2%)	0.011
Hepatomegaly	14 (18.7%)	4 (14.8%)	0.653
**Concomitant disease (in anamnesis)**			
Prior myocardial infarction	34 (45.3%)	8 (29.6%)	0.155
Hypertension	52 (69.3%)	16 (59.3%)	0.341
Atrial fibrillation	45 (60.0%)	9 (33.3%)	0.017
Previous stroke or transient ischemic attack	6 (8.0%)	3 (11.1%)	0.625
Diabetes mellitus (type 2)	40 (53.3%)	10 (37.0%)	0.146
Chronic obstructive pulmonary disease	12 (16.0%)	3 (11.1%)	0.539
Chronic kidney disease (stage ≥ 3)	27 (36.0%)	3 (11.1%)	0.014
**Pharmacotherapy**			
ACE-I	53 (71.6%)	9 (34.6%)	0.0008
ARB	7 (9.5%)	3 (11.5%)	0.761
Beta-blockers	66 (89.2%)	12 (46.2%)	<0.0001
MRA	30 (40.5%)	3 (11.5%)	0.007
Diuretics	65 (87.8%)	9 (34.6%)	<0.0001
Laboratory tests			
Creatinine (mg/dL)	1.30 (1.00–1.60)	1.10 (0.80–1.40)	0.030
eGFR, (mL/min/1.73 m^2^)	56.4 (41.8–70.7)	72.2 (53.2–90.2)	0.007
NTproBNP (pg/mL)	3640 (1509–7102)	4902 (2512–8563)	0.258
High-sensitivity troponin T (ng/L)	38.8 (24.5–57.6)	30.5 (19.3–67.3)	0.371
WBC (109/L)	7.5 (6.4–9.1)	8.5 (7.5–11.4)	0.001
Hemoglobin (g/dL)	12.4 (10.9–13.5)	14.0 (12.6–15.2)	0.004
Anemia	49 (65.3%)	8 (29.6%)	0.001
Bilirubin (mg/dL)	1.00 (0.6–1.7)	1.0 (0.8–1.5)	0.683
ALT (IU)	31.0 (21–38)	30.0 (25–52)	0.142
AST (IU)	21 (15–34)	33 (28–47)	0.037
Hospitalization			
Days of hospitalization	8 (6–13)	8 (7–11)	0.965
Total IV dose of loop diuretics (mg)	580 (340–900)	380 (320–1000)	0.442
Total oral dose of loop diuretics (mg)	280 (160–480)	320 (120–400)	0.806
**Hemodynamics at admission**			
LVEF (%)	33 (25–50)	35 (30–50)	0.593
HR (bpm)	76 (65–86)	87 (65–111)	0.029
Systolic BP (mmHg)	118 (103–135)	122 (114–149)	0.183
Diastolic BP (mmHg)	70 (66–80)	81 (72–85)	0.029
SI (mL*m^−2^)	41.1 (33.0–47.8)	36.0 (25.6–43.0)	0.043
CI (mL*m^−2^*min^−1^)	3.0 (2.40–3.6)	2.9 (2.6–3.3)	0.377
SVRI (dyn*s*cm^−5^*m^2^)	2115 (1671–2603)	2604 (2142–3044)	0.013
TFC (1*kΩ^−1^)	33.6 (29.3–39.3)	37.8 (31.3–41.9)	0.194
TFC ≥ 35.1*kΩ^−1^	33 (44.0%)	15 (55.6%)	0.302

ACE-I—angiotensin-converting enzyme inhibitor, ADCHF—acutely decompensated chronic heart failure, ALT—aspartate transaminase, ARB—angiotensin receptor blockers, AST—aspartate aminotransferase, BMI—body mass index, BP—blood pressure, CI—cardiac index, dnHF—de novo heart failure, eGFR—estimated glomerular filtration rate, HR—heart rate, LVEF—left ventricle ejection fraction, MRA—mineralocorticoid receptors antagonist, NTproBNP—N-terminal pro-brain natriuretic peptide, NYHA—New York Heart Association, SI—stroke index, SVRI—systemic vascular resistance index, TFC—thoracic fluid content, WBC—white blood cells.

**Table 3:** Comparison of discharge characteristics between patients hospitalized with acutely decompensated CHF (ADCHF) and de novo HF (dnHF).

	ADCHF Median (IQR); n (%)	dnHF Median (IQR); n (%)	p
**Change from admission to discharge**			
NTproBNP (pg/mL)	1061 (216–2794)	2393 (1245–4627)	0.026
TFC (1*kΩ^−1^)	3.8 (0.8–8.8)	7.8 (2.4–13.3)	0.013
Body mass (kg)	3.8 (1.5–6.3)	4.9 (3.4–9.6)	0.228
**Hemodynamics at discharge**			
HR (bpm)	72 (63–81)	81 (73–103)	0.002
Systolic BP (mmHg)	109 (94–122)	107 (94–115)	0.436
Diastolic BP (mmHg)	67 (60–74)	68 (62–75)	0.489
SI (mL*m^−2^)	40.5 (33.4–48.5)	29.5 (27.0–37.0)	<0.0001
CI (mL*m^−2^*min^−1^)	2.9 (2.5–3.7)	2.7 (2.2–3.0)	0.023
SVRI (dyn*s*cm−5*m^2^)	2023 (1512–2390)	2201 (1842–2596)	0.087
TFC (1*kΩ^−1^)	29.6 (25.9–34.7)	28.6 (23.1–32.8)	0.358
	**Pharmacotherapy**		
ACE-I	62 (86.1)	23 (85.2)	0.906
ARB	4 (5.6)	1 (3.7)	0.708
Beta-blockers	70 (97.2)	26 (96.3)	0.811
MRA	41 (56.9)	19 (70.4)	0.223
Diuretics	71 (98.6)	27 (100.0)	0.538

## Data Availability

The data presented in this study are available upon request from the corresponding author. The data are not publicly available due to privacy or ethical restrictions.
